# RiboScreen^TM^ Technology Delivers a Ribosomal Target and a Small-Molecule Ligand for Ribosome Editing to Boost the Production Levels of Tropoelastin, the Monomeric Unit of Elastin

**DOI:** 10.3390/ijms25158430

**Published:** 2024-08-01

**Authors:** Bjoern Wimmer, Jan Schernthaner, Genevieve Edobor, Andreas Friedrich, Katharina Poeltner, Gazmend Temaj, Marlies Wimmer, Elli Kronsteiner, Mara Pichler, Hanna Gercke, Ronald Huber, Niklas Kaefer, Mark Rinnerthaler, Thomas Karl, Jan Krauß, Thomas Mohr, Christopher Gerner, Helmut Hintner, Michael Breitenbach, Johann W. Bauer, Christin Rakers, Daniel Kuhn, Joerg von Hagen, Norbert Müller, Adriana Rathner, Hannelore Breitenbach-Koller

**Affiliations:** 1 Department of Biosciences and Medical Biology, University of Salzburg, 5020 Salzburg, Austria; bjoern.wimmer@stud.plus.ac.at (B.W.); jan.schernthaner@ursprung.at (J.S.); genevieve.edobor@plus.ac.at (G.E.); andreas.friedrich@sbg.ac.at (A.F.); kathi.poe@gmail.com (K.P.); marlies.wimmer@stud.plus.ac.at (M.W.); elli.kronsteiner@stud.plus.ac.at (E.K.); mara.pichler@stud.plus.ac.at (M.P.); hanna.gercke@plus.ac.at (H.G.); ronald.huber@stud.plus.ac.at (R.H.); mark.rinnerthaler@plus.ac.at (M.R.); thomas.karl@plus.ac.at (T.K.); krauss@skm-ip.de (J.K.); michael.breitenbach@plus.ac.at (M.B.); 2 Human Genetics, Faculty of Pharmacy, College UBT, 10000 Pristina, Kosovo; gazmend.temaj@ubt-uni.net; 3 Department of Analytical Chemistry, Faculty of Chemistry, University of Vienna, 1090 Vienna, Austria; thomas.mohr@univie.ac.at (T.M.); christopher.gerner@univie.ac.at (C.G.); 4 Join Metabolome Facility, University of Vienna, Waehringer Str. 38, 1090 Vienna, Austria; 5 Department of Dermatology and Allergology, University Hospital Salzburg, Muellner Hauptstraße 48, 5020 Salzburg, Austria; hhintner@icloud.com (H.H.); joh.bauer@salk.at (J.W.B.); 6 Merck KGaA, Discovery & Development Technologies, Frankfurter Staße 250, 64293 Darmstadt, Germanydaniel.kuhn@merckgroup.com (D.K.); 7 Merck KGaA Healthcare, Frankfurter Straße 250, 64293 Darmstadt, Germany; joerg.von.hagen@merckgroup.com; 8 ryon-Greentech Accelerator, Mainzer Straße 41, 64579 Gernsheim, Germany; 9 Institute of Biochemistry, Johannes Kepler University, Altenbergerstraße 69, 4040 Linz, Austria; norbert.mueller@jku.at; 10 Department of Chemistry, Faculty of Science, University of South Bohemia in Českých Budějovicích, Branišovská 1760, 370 05 České Budějovice, Czech Republic

**Keywords:** tropoelastin expression, elastin, ageing of elastic tissues, ribosomal protein eL40, small-molecule hit, customized protein expression, RiboScreen^TM^ Technology

## Abstract

Elastin, a key structural protein essential for the elasticity of the skin and elastogenic tissues, degrades with age. Replenishing elastin holds promise for anti-aging cosmetics and the supplementation of elastic activities of the cardiovascular system. We employed RiboScreen^TM^, a technology for identifying molecules that enhance the production of specific proteins, to target the production of tropoelastin. We make use of RiboScreen^TM^ in two crucial steps: first, to pinpoint a target ribosomal protein (TRP), which acts as a switch to increase the production of the protein of interest (POI), and second, to identify small molecules that activate this ribosomal protein switch. Using RiboScreen^TM^, we identified ribosomal protein L40, henceforth eL40, as a TRP switch to boost tropoelastin production. Drug discovery identified a small-molecule hit that binds to eL40. In-cell treatment demonstrated activity of the eL40 ligand and delivered increased tropoelastin production levels in a dose-dependent manner. Thus, we demonstrate that RiboScreen^TM^ can successfully identify a small-molecule hit capable of selectively enhancing tropoelastin production. This compound has the potential to be developed for topical or systemic applications to promote skin rejuvenation and to supplement elastic functionality within the cardiovascular system.

## 1. Introduction

Elastin, an extracellular matrix (ECM) protein, is the major contributor to the elasticity of extensible tissues, the skin, the arteries of the cardiovascular system, the lung, cartilage and tendons. Thus, tropoelastin (TE) the precursor of elastin, is synthesized in fibroblasts, smooth muscle cells, chondrocytes or endothelial cells [1]. Elastin is deposited in elastic fibers and interacts with tissue specific ECM proteins to customize the stretch and coil cycles of the individual elastic tissues. For example, in the skin, elastin, in combination with subcutaneous fat and an array of cutaneous extracellular matrix proteins, such as proteoglycans and glycosaminoglycans, is required for maintaining healthy skin structure and function [2,3].

Elastic fibers are assembled through a process known as elastogenesis, which takes place in elastogenic cell types, such as skin fibroblasts. This is dependent on the expression, hierarchal assembly, and crosslinking of elastin´s dominant component, tropoelastin [4,5]. Elastogenesis uses soluble tropoelastin monomers, which are secreted by and bound to fibroblasts receptors. There, tropoelastin aggregates into microscopic globules, a process termed coacervation. During this process, tropoelastin molecules are oxidized by different members of the lysyl oxidase family. This induces crosslinking to form the elastin polymer. Elastin then associates with fibrillin-rich microfibrils to form a nascent elastic fiber that is released from the cell surface into the surrounding milieu of the extracellular matrix [6].

Elastin is a remarkably long-lived protein and is primarily deposited during prenatal development and childhood, with little turn-over, and in skin, the overall half-life of elastin is similar to human life span [7]. However, moderate neo-synthesis of the monomeric precursor tropoelastin occurs in adult and aged skin cells [8,9], and early studies showed that steady-state, basal levels of elastin production and breakdown are observed in adult tissues as well as in cells isolated from adult tissues [10,11,12].

The degradation of elastic fibers in all elastic tissues starts with advancing age, and in the skin, exposure to environmental insults causes additional loss of functional elastic fibers and thus structural integrity of the skin. When combined with subcutaneous fat loss, this results in looser, sagging skin, causing undesirable changes in appearance. In parallel, damaging changes occur in chronically sun-exposed skin, which shows deregulation in amount and arrangement of cutaneous elastic fibers and loss of fine elastic fibers in the superficial dermis, required for a flexible connection to the epidermis. Indeed, the most devasting effect on skin elasticity results from the replacement of the normal collagen-rich superficial dermis with abnormal clumps of solar elastosis material formed upon prolonged exposure to UV light [13,14]. In an analogous fashion, the disruption of elastic fiber networks after injury and during wound healing also leads to undesirable characteristics in the structure and appearance of scars and stretch marks. During wound closure, abnormally arranged, large bundles of collagen are deposited to promote wound closure. While injury also induces the reinitiation of tropoelastin expression [3], elastic fibers are the last ECM fibers that arrive at the scene during the remodeling phase of the scar tissues and are not produced at the required levels to establish the reformation of the elastic fiber network to provide the scar tissue with elasticity comparable to the original tissue.

Prior to discussing present and future strategies to replenish tropoelastin monomers and subsequently elastin and elastic fibers, we will review the transcriptional and posttranscriptional regulation of the *ELN* gene, which encodes tropoelastin. The human *ELN* gene harbors 34 in-frame exons, and alternative splicing of the *ELN* pre-mRNA generates, without the alteration of the open reading frame, a suite of mature mRNA isoforms for the translation of tropoelastin [15]. Still unresolved is the enigma of continuous *ELN* mRNA production in the post-adolescent period, long after the protein production of tropoelastin is at a minimal level. Two regulatory operators might work in concert to orchestrate this phenomenon. Therefore, first, the promotor structure of the *ELN* gene as a landing platform for different transcription factors is discussed, and second, we put the spotlight on the control of *ELN* mRNA half-life. An excellent review by Procknow and Kozel [16] provides recent insights into these complex, multistep transcriptional and posttranscriptional regulations of tropoleastin production.

The *ELN* promotor has been characterized within a sequence tract of 2.2 kb upstream of the initiating methionine and exhibits the characteristics of a conventional “housekeeping” gene, which is ubiquitously and continuously transcribed, i.e., a GC-rich sequence, the absence of a conventional TATA box, and the presence of multiple transcriptional start sites, organized in a defined orientation [17]. First, and most proximal to the initiating methionine resides in a CAAT box regulatory site, targeted by the C/EBPβ (CCAAT/enhancer-binding protein beta) transcription factor to regulate *ELN* transcription during inflammation and tissue remodeling. The activity of C/EBPβ is context-dependent; for example, in the case of binding of pro-inflammatory cytokine IL-1β (Interleukin-1 β) to C/EBPβ, *ELN* expression is decreased. This is in line with reports that NF-κB (nuclear factor kappa B) in concert with IL-1ß decreases *ELN* transcription in response to inflammatory signals [18]. Second, multiple GC boxes are present, which in the *ELN* promotor serve as binding site for the Sp1 (Specificity Protein1) member of the Sp transcription factor family and, together with activated Rb (Retinoblastoma), promote the transcription of *ELN* in response to TGFβ1 (Transforming Growth Factor beta 1) and IGF1 (insulin-like growth factor 1). Again, the effect of TGFβ1 is highly context-dependent, varying between cell types and cellular states. On the other hand, the transcription of *ELN* is inhibited when cytokines IL1β and PDGF (Platelet-Derived Growth Factor) disrupt Sp1 binding to the GC box. Third, there are several AP_1 (Activator Protein 1) binding sites, providing docking sites for the Jun and Fos class of transcription factors. Members of this transcription factor family form homodimers and heterodimers to generate the AP1-Complex, whose binding to the regulatory AP1 site is under the control of different cytokines. For example, key inflammatory cytokine TNFβ (tumor necrosis factor beta) promotes the binding of a Jun homodimer to proximal AP1 site, thereby inhibiting *ELN* transcription (Proknow). Fourth, there are several GRE (Glucocorticoid Response Element) promoter sequences, which accept dimers of the activated GR (glucocorticoid receptor) to promote *ELN* expression. A set of putative additional transcription factor binding sites in the more extended *ELN* promotor is predicted by the ENCODE project [19], and the JASPER CORE collection database [20]. Among these factors, DNA binding proteins were found, which regulate the transcriptional machinery’s access to the coding region of the gene, for example, histon-modifying complexes and CTCF (CCCTC-binding factor). CTFN is a multifunctional protein for which roles in insulator activity, the remodeling of chromatin architecture, and the regulation of RNA splicing have been described (10.1098/rstb.2012.0369). This multi-layered scenario of the transcriptional control of the *ELN* mRNA provides a rationale for the fine-tuned regulation of *ELN* expression in the development and different tissues to ensure the delivery of tropoleastin at appropriate levels but does not account for the scant production levels of tropolelastin protein in mature tissues. The second major factor to determine the final level of tropoelastin as produced by *ELN* mRNA in mature tissue has been attributed to a rapid decline in the half-life of mature *ELN* mRNA [21]. MicroRNAS (miRNAS), which are short, noncoding RNA molecules that target mRNA to decay, have been implicated in the regulation of *ELN* mRNA abundance. In particular, members of the miR-29 family of miRNAS have been shown to modulate *ELN* mRNA steady-state concentration [22]. However, miR-29 family members are not specific to the regulation of *ELN* mRNA abundance as they also target other ECM mRNASs, for example, COL1A1 (encoding pro-alpha1 chains of type I collagen) and COL3A1 (encoding alpha-1 chain of type III collagen) [23]. This lack of specificity limits the use of miR-29 family members as attractive therapeutic targets.

There is a great medical–aesthetic need for interventions to replenish tropoelastin and consequently elastin and elastic fibers: first, to improve wound healing capacity; second, to enhance the skin´s texture and resilience during the aging process; and third, to combat the insufficiency of functional elastin in cardiovascular and pulmonary diseases [1,3,24,25,26,27]. Several ingenious strategies have been developed to substitute tropoelastin and elastin in skin and other tissues. Halawani et al. used implantation regimes to study the regenerative and wound-healing purposes of supplemented tropoelastin and studied the role of tropoelastin augmentation in accelerating wound repair by enhancing vascularization at the site of implantation [28]. Golombeck et al. demonstrated in vivo increased dermal TE synthesis by the exogenous administration of synthetic mRNA [29]. Kellar et al. used a tropoelastin and collagen wound-healing device to slowly release these protective molecules in a diabetic mouse and observed, in comparison to untreated control, improved regeneration of the skin organ, an enhanced rate of wound closure, decreased tissue inflammation, and stronger and more durable remodeled tissue [30]. Mithieux et al. developed a synthetic material whose active component is tropoelastin and which is used as a tailored insert shaped to the wound bed [31]. These authors demonstrated in a porcine model the benefit of their intervention with respect to the extent of wound healing, dermal repair, and regeneration of the mature epithelium. These different strategies for tropoelastin supplementation, while ingenuous, share one disadvantage, namely the necessity of an exogenous supply of tropoelastin. Alternatively, a targeted endogenous increase of the protein production level of tropoelastin within the skin and other elastic tissues would be desirable.

The primary objective of this study was to utilize RiboScreen^TM^ Technology [32,33,34], which enables the delivery of a potent small molecule, which, by selectively binding to a target ribosomal protein (TRP), subsequently tailors the production level of tropoelastin. We report ribosomal protein eL40 as a TRP for tuning tropoelastin levels and deliver a small-molecule ligand of eL40, which in cell assays is shown to boost protein production levels of tropoelastin two-fold. Overall, we present an alternative strategy to supplement tropoelastin in aging tissues or, during wound healing, providing a directed boost of tropoelastin expression at a physiological level as induced by a small molecule.

## 2. Results and Discussion

### 2.1. Considerations Regarding RiboScreen^TM^ Technology (Assay Principle)

**Figure 1:**
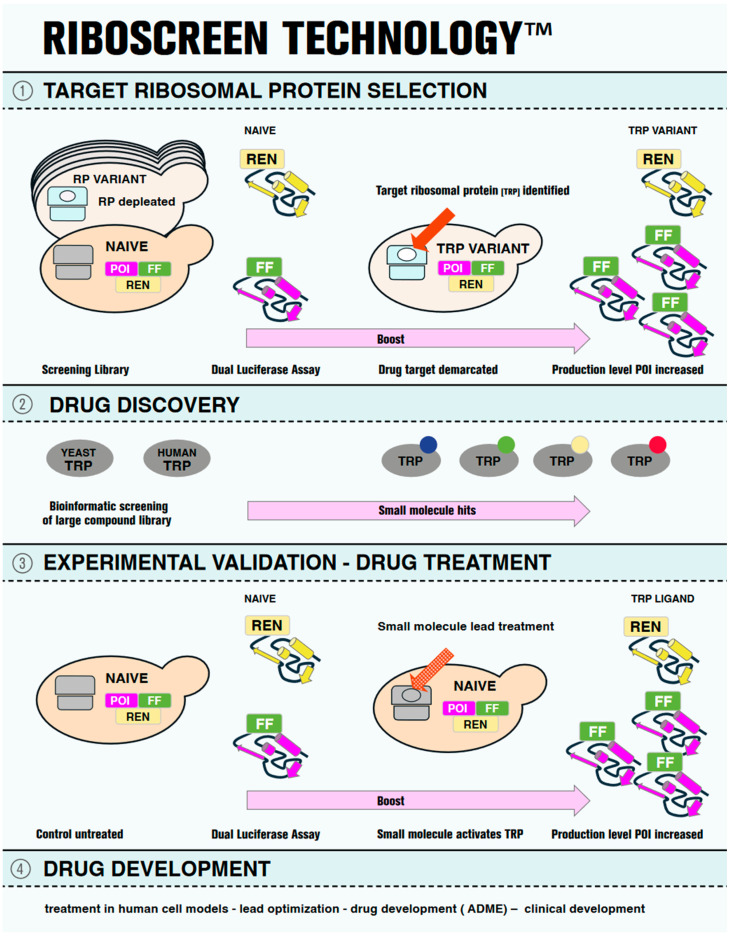
Pictographic representation of RiboScreen^TM^ Technology. On the left-hand side, the first step of the RiboScreen^TM^ technology is shown in Panel (1). Two tools are used. First is a screening library of yeast vehicles, each of which is depleted for one of the eighty eukaryotic ribosomal proteins (RPs), the ribosomal variant strain (RVS) screening library. In cyan, a depleted ribosome for a ribosomal protein (white oval) is shown. In grey, a wild-type ribosome is shown in a naïve vehicle. The second tool is a dual luciferase assay to monitor protein expression levels of the protein of interest (POI, magenta), the cellular target. The reporter of the POI carries a C-terminally tagged Firefly luciferase (FF, green), and a Renilla (REN, yellow) luciferase reporter serves as internal control. In the middle section, panel (1) presents the identification of the actual drug target, the target ribosomal protein (TRP) (red arrow), demarcated by its altered functional availability (white oval), which leads to an increase in the production level of the POI, (panel (1), right). Panel (2) shows orthologous yeast and human TRPs (grey ovals) on the left, which serve as protein baits for structure-based virtual screening to identify small-molecule binders. The compounds identified in this way from the screening of a library of small molecules are then computationally docked into the binding sites of the TRP and scored for their binding affinities to identify potential ligands. The virtual screening results are post-processed to select the representative hits (small colored circles) for further analysis (panel (2), left). Panel (3) shows the experimental validation of the hit molecules using RiboScreen^TM^ technology. Naïve vehicles, equipped with identical protein reporters as employed in the initial screening (POI-FF and REN), are used (Panel (3), left), but here treated with TRP ligands (red dotted arrow). Hit compounds are identified based on their demonstrated activity to boost POI production levels. In Panel (4), further steps of drug development are listed for completeness.

RiboScreen^TM^ technology is a pipeline technology that provides, in a stepwise fashion, first the target ribosomal protein (TRP), which controls the production level of a protein of interest (POI); second, small-molecule ligands of the TRP; and third, functional in-cell assays to detect TRP ligand activity in endogenously modulating the protein production levels of POI (Figure 1). In other words, this is a “chain” technology, where the small molecule is the effective compound that targets the TRP (first in the chain), which then modulates the production level of the POI (second in the chain), all within a physiological range and therefore with minimal off-target effects [32,33,34]. Also, for RiboScreen^TM^ technology, the aspect of a POI being “undruggable” is irrelevant, as this innovative technology targets not the POI, but the production level of the POI. This is truly a stand-alone protein rescue technology inspired by nature. 

To offer a more detailed view of RiboScreen^TM^ technology, we describe the individual components of this assay principle here:

- The ribosome and the concept of specialized ribosomes.
- The yeast screening library employed for the identification of a TRP specific for the translational control of a POI.
- The dual luciferase assay to monitor protein expression levels of reporter proteins in the screening library.
- The search for and characterization of a TRP ligand.
- The application of the dual luciferase assay in naïve cellular vehicles to detect endogenous small-molecule effects of the TRP ligand in enhancing the production levels of the POI.

The ribosome is a two-subunit molecular translator of mRNA in all kingdoms of life. The translating ribosome is assembled from two unequal subunits, each composed of ribosomal RNA (rRNA) and ribosomal proteins (RPs) [35]. The more complex eukaryotic ribosome consists of a large 60S subunit harboring 50 ribosomal proteins and a small 40S subunit harboring 30 ribosomal proteins [36,37,38]. While rRNA tracts have been expanded during eukaryotic evolution, the 80 ribosomal proteins of the eukaryotic cytoplasmic ribosome have been highly conserved from yeast to humans in sequence, structure, and ribosome topology and bind to homologous rRNA sequence tracts [37,38]. Based on these observations, ribosomal proteins have been investigated across a range of model organisms to investigate their possible regulatory role in the eukaryotic translational control of gene expression [39].

Also, within the last two decades, the development of advanced techniques in several model organisms has allowed the identification of a heterogeneity of ribosome complements with respect to their composition and modification of ribosomal proteins, respectively [40,41,42]. In particular, ribosomal proteins were shown to differ in stoichiometry and posttranslational modifications between different cell types and metabolic states, suggesting a reservoir of ribosomal protein variants as a tool kit for generating heterologous ribosomes [43]. Several lines of evidence now corroborate the potential of a heterologous sub-population of ribosomes to function as specialized ribosomes [44]. This specialization of ribosome function is thought to promote intrinsically altered translational activity, which is tailored to change the production level of a particular protein or a selected set of functionally related proteins only [43,45], while leaving bulk protein synthesis unaltered [32,44].

As such specialized ribosomes in nature are generated by modifications of ribosomal constituents, ribosomal proteins are of particular interest for the bioengineering of specialized ribosomes. Their high structural and functional conservation during eukaryotic evolution allows for the development of cost- and time-efficient screening assays in yeast vehicles. 

Therefore, the yeast Saccharomyces cerevisiae ribosome with its constituent ribosomal proteins serves as a prototypic model for exploiting natural strategies to engineer specialized ribosomes [32].

RiboScreen^TM^ technology employs a large collection of diploid yeast strains as a screening tool, which generates heterozygosity for individual ribosomal proteins (RPs) by heterozygous ribosomal protein gene deletions in one or the other of the 80 eukaryotic cytoplasmic ribosomal proteins [23]. Within this ribosomal variant protein strain (RVS) collection, each RVS carries distinct sub-populations of altered, heterologous ribosomes, demarcated by the absence of a distinct RP. Such an altered state of functional availability of a ribosomal protein serves as proxy for testing the ribosomal protein-regulatory activity for the tailored production levels of a POI [23].

Protein production levels are quantified through comparative protein synthesis assays, utilizing a dual luciferase assay that measures the expression level of a C-terminal Firefly (FF) tagged POI, in this study a tropoelastin reporter (TE-FF) and an independently expressed Renilla (REN) luciferase control reporter. Through rigorous statistical analysis of protein production levels observed in the RVS screening library, a variant is identified, which is demarcated by the reduced availability of the target ribosomal protein (TRP), thereby tuning the production level of the protein of interest (POI-FF), e.g., TE-FF, without affecting the control (REN) (Figure 1, Panel 1).

For TRP ligand screening, industry cooperation may be employed, which uses TRP as template for virtual screening (docking) that could deliver small-molecule hits. Alternatively, literature search and content screening may also deliver TRP ligands (Figure 1, Panel 2).

In the next step, the positively screened small-molecule ligands of TRP are tested in yeast wild-type vehicles to query their potential as modulators of the TRP function in increasing the production of the POI (Figure 1, Panel3). Hits that exhibit in vivo activity (in our experience, only few of the hits) serve as hit compounds that are further developed in human and animal cellular models (Figure 1, Panel 4).

We wish to complement these considerations by reporting on the testing compounds identified with the RiboScreen^TM^ tool for their potential activity in human cell models. First, in our prototypic example for RiboScreen^TM^ technology employing a yeast-vehicle-based screening assay, we identified rpL35 as a target ribosomal protein (TRP) to boost the production levels of human reporter protein Lamb3, a skin anchor protein [32]. Second, we identified two small molecules, Artesunate and Atazanavir, which bind to yeast and human rpL35 [33]. Third, we showed the activity of Artesunate and Atazavir in boosting Lamb3 production levels, both in yeast vehicles, as individual drugs and in combinatorial use at a reduced dosage (Wimmer et al., 2024), as well as in human model cells [46]. It is important to note that RiboScreen^TM^ technology delivers pre-screened small molecules for targeted treatment in a human cell model, in which a small-molecule targets a human TRP (rPl35) for customized increase at a physiological level (two-fold) in the production level of POI (Lamb3). This is achieved with minimal interference to the protein production of bulk protein synthesis, thereby minimizing off-target effects, for example, interference with the transcriptional machinery or overload of the downstream processes of protein secretion. Also, the primary drug response, i.e., tuning protein production level of the POI (Lamb3), no longer warrants a reporter protein assay but can be monitored directly to quantify protein levels of the POI (Lamb3) with proteomic or Western blot analysis. Following this rationale, the treatment of human cell models with small molecules to boost tropoelastin expression are expected to also avoid the pitfalls of non-targeted approaches.

### 2.2. Tropoelastin Isoform 6 as a Luciferase-Based Protein Production Reporter

**Figure 2:**
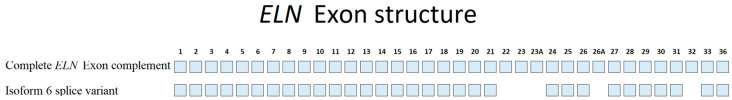
Exon structure of ELN mRNA encoding Tropoelastin. On top, the ELN exon structure is depicted, with the individual exons numbered as present in the human mRNA (modified from [47]). The bottom shows isoform 6 splice variant, lacking several exons, among them exon 26A.

Tropoelastin (TE), the subunit of the elastic fiber, is a soluble 60–70 kDa protein and is encoded by the *ELN* gene, which in the human genome is located on the long arm of chromosome 7q11.2 [1]. Human ELN gives rise to a broad variety of splice isoforms [47]. Indeed, variations in the relative abundance of alternatively spliced ELN mRNA transcripts and their corresponding protein isoforms have been documented between tissues, and this diversity is thought to be necessary for the fine tuning of elastogenesis to confer the unique functional mechanical characteristics to different tissues [48]. Variations in isoform production have also been reported in certain disease states and photo aging, which induces unscheduled tropoelastin production, specifically from an isoform containing exon 26A. This isoform is not upregulated upon treatment with retinoic acid, a topically applied skin rejuvenation regime, which increases the production of tropoelastin isoforms lacking exon 26A [49], accompanied by a beneficial increase of functional elastin [50,51]. However, prolonged therapy using retinoids, all of which are derivatives of Vitamin A, is associated with irritant reactions such as burning, scaling, or dermatitis, limiting their acceptance by patients. Together, these observations encouraged us to use as a reporter protein for this study a tropoelastin isoform lacking exon 26A (Uniprot ID P15502-6) and which is commercially available [52], i.e., isoform 6 (Figure 2).

### 2.3. RiboScreen^TM^ Identifies Ribosomal Protein eL40 as a Candidate Target Ribosomal Protein for Boosting Tropoelastin Protein Production

Here, we used a library of 137 yeast strains derived from the yeast diploid BY4742 wild-type strain [32,53], with each strain being heterozygous for ribosomal protein gene deletion in one or the other of the 80 eukaryotic cytoplasmic ribosomal proteins. This library includes heterozygous gene deletions of paralogous ribosomal protein genes, which are present for two-thirds of all yeast ribosomal proteins [54]. Therefore, each strain serves as a ribosomal variant strain (RVS) vehicle, which is expected to have a sub-population of altered ribosomes as a consequence of reductions in RP gene dosage of a particular RP (Figure 1, panel 1), with the remaining ribosomes being of the wild type [55]. For screening purposes, this should provide minimal interference with overall ribosome function, comparable to the modification of a ribosomal protein by post-translational modification in nature [42,54,56]. Our approach excludes the use of RP gene deletions in haploid yeast vehicles, as these would generate ribosomes that are completely devoid of a given ribosomal protein, a condition that is not compatible with growth for most haploid RP gene deletions [53]. However, also in the diploid condition, the reduction in some single copy RPs, as well as the reduction in one RP protein paralogue, may reduce the cellular viability of yeast vehicles [55]. This was also observed in this screen, and out of 138 screening strains, including the WT strain, 9 strains were not viable under the culture conditions used in this study and therefore were not included in this screening. These were RVS vehicles depleted for ribosomal proteins encoded by ribosomal protein genes RPL4B, RPL5, RPS8B, RPL9B, RPL11A, RPL14B, RPS26A, RPL31B, and RPL36B and which also did not grow in our previous screens [32]. This leaves 128 RVS and one WT screening vehicle for this study.

From the RVS screening library, we intended to identify an RVS characterized by the reduction in a specific ribosomal protein that specializes the ribosome for customized change in production levels of a tropoelastin reporter protein while leaving the production levels of a companion control reporter unaltered. First, one reporter was generated using TE isoform 6, which was C-terminally tagged with a Firefly (FF) luciferase tag (TE-FF).

A second, companion reporter plasmid, expressing the Renilla (REN) luciferase reporter, was used as an independently expressed control protein [32]. This luciferase reporter pair is engineered to harbor identical 5´ and 3´ regulatory untranslated regions (UTRs), which are derived from yeast ADH1 mRNA and harbor regulatory sequences mediating robust translation initiation and termination, respectively [32,57].

In the next step, after the co-transformation of the reporter pair and following our established screening protocol [32], reporter protein luciferase signals of the TE-FF and REN reporter pairs were recorded in each of the RVS screening vehicles. After outlier analysis, a statistical method that operates under the assumption that the collected data are drawn from a larger normal distribution, the descriptive statistical analysis of the data set was performed, which is reported in Supplementary Table S1. To reduce the complexity of dual luciferase readout data obtained in the collection of RVS vehicles, a Pearson product moment (ρ) was generated to query the variation in protein production levels of companion reporters TE-FF and REN within the RVS screening library. A value of ρ = 0.64 was calculated, which indicates for the majority of RVS vehicles a similar protein production level of TE-FF and REN reporters. This value, ρ = 0.64, does not say anything about whether these similar production levels of the companion reporters are higher or lower than for wild-type vehicles.

**Figure 3:**
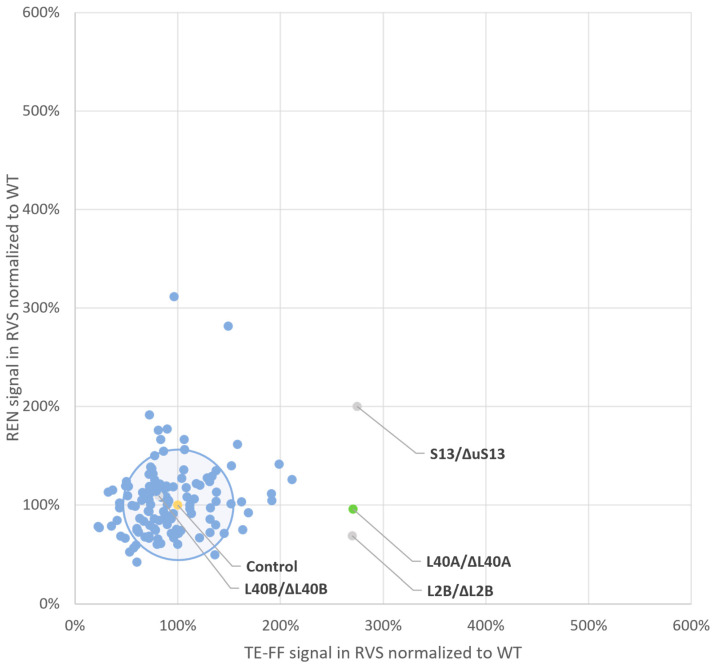
Reporter protein production levels in the ribosomal variant strain (RVS) screening library. This plot correlates, in a single data point for each ribosomal variant strain vehicle, the mean protein production level of the internal control REN reporter, normalized to the mean wild-type signal on the *y*-axis and the mean protein production level of the TE-FF reporter normalized to the mean wild type signal on the *x*-axis. A circle centered at a 100% expression level (i.e., 1) of both reporter proteins in the wild-type vehicle (marked in yellow) and with a radius of 50% difference in expression level, corresponding to the 2-fold standard deviation of the overall mean of measurements in all RVS contains the TE-FF and REN reporter expression profiles from the majority of screening vehicles. The data point obtained for the RVS, carrying a depletion for eL40, as encoded by RPL40A (marked in green), but not as encoded by RPL40B (marked in grey), signals that the modification of this functional availability of eL40 boosts TE-FF production 2.8-fold while leaving REN expression levels unaltered. This identifies eL40 as a potential target ribosomal protein (TRP) for customized boost of protein production levels of tropoelastin. Minor TRP species, where REN expression levels are also affected, are represented by the reporter protein expression levels observed in RVS depleted for ribosomal proteins uS15 and uL2 as encoded by RPS13 and by RPL2B (marked in grey). Note that the gene nomenclature and protein nomenclature are different for ribosomal proteins in yeast [40].

To obtain more insight into the distribution of companion protein reporter expression levels and to visualize these co-expressed luciferase signals in each of the 129 screening vehicles (128 RVS and 1 WT), we used a correlation plot. Accordingly, for each RVS vehicle investigated, the mean TE-FF expression level normalized to the mean WT expression level was plotted against the respective mean companion REN expression level normalized to the mean WT expression level (Supplementary Table S1, Figure 3). Thus, the production levels of the accompanying reporters are recorded in a single data point in the correlation plot for each RVS vehicle. It is important to note that, representing as one data point (blue dots in Figure 3) the normalized luciferase readouts of the two companion reporters, TE_FF (on the *x*-axis) and REN (on the *y*-axis), as obtained in each one of the screening vehicles of the RVS library, generate a composite image of the complete RVS screen.

As shown in Figure 3, most RVSs, defined by the heterozygous depletion of a ribosomal protein, exhibited minimal changes in the protein expression levels of either reporter. Furthermore, the reporter expression from RVSs with minimal changes in protein production closely resembled wild-type levels. In Figure 3, these RVS clusters within a circle representing a 0.5-fold difference in protein production were compared to the wild type. The circle’s radius is equivalent to the overall two-fold standard deviation of normalized wild-type TE-FF measurements (48%, Supplementary Table S1). This circle of “inert” RVSs with minimal protein production changes encompasses 80% (104 out of 129) of the screened vehicles. Conversely, most RVSs (25 out of 129) that exhibit a greater than 0.5-fold change in protein production compared to the wild type for either reporter localize outside this circle in Figure 3. These RVSs are distributed across the four quadrants of a square defined by a two-fold increase or decrease in protein production relative to the wild type. 

There are such RVS vehicles that either decrease both reporters (clockwise, quadrant 4) or increase both reporters (quadrant 2) and thus also follow the ρ = 0.64 Pearson product moment. Also, there are RVSs that preferably generate an increase in either the REN signal (quadrant 1) or the TE-FF signal (quadrant 3). However, we aimed to identify an RVS vehicle demarcated by a ribosomal protein depletion that selectively increases the TE-FF protein production levels while leaving REN production levels unaltered. The inspection of Figure 3 shows that such a candidate specialization of ribosomes might be generated by an RVS carrying a depletion of ribosomal protein eL40 as encoded by the RPL40A gene. It is important to note that only this RVS, as demarcated by reduced functional availability of eL40, delivers a tailored 2.7-fold (170%) increase in TE-FF production levels (*p* = 0.95), i.e., the POI, with no change in the REN expression level (*p* = 0.95) (Supplementary Table S1), and with no other RVS vehicle to deliver such a tailored response.

While eL40 is a promising TRP candidate due to its specific impact on TE-FF production (Figure 3), two RVSs depleted for ribosomal proteins, uS15 and uL2, as encoded by the RPS13 and RPL2B genes, respectively, exhibited increased TE-FF levels that were similar to eL40 depletion. However, these RVSs also displayed altered REN expression upon the depletion of the ribosomal proteins uS15 and uL2, respectively. Therefore, to isolate the effect on TE-FF, we excluded them from further analysis.

We note that the RVS vehicle carrying a reduction in the eL40 protein as encoded by the RPL40B paralog is inert to the changing expression levels of either TE-FF or REN (Figure 3). The phenomenon of the differential function of the paralogous ribosomal proteins is thought to result from their different expression level, which can be tuned to different cellular states [55,58].

Although ribosomal proteins can be expressed by different genetic loci during ribosome biogenesis, a specific protein can only occupy its designated binding site on the mature ribosome. This concept suggests that differential expression levels of ribosomal protein paralogs could be a natural mechanism for generating specialized ribosomes with distinct translation properties [55]. Interestingly, genome-wide expression data suggest that RpL40A might contribute about 60% and RpL40B might contribute about 40% of the total mRNA levels to be translated into eL40 [59]. Therefore, while either eL40 paralog, when highly expressed, can functionally substitute for the other paralogue to sustain growth [59], heterozygosity for RpL40A, but not that RPL40B, might promote a threshold effect to specialize ribosomes to boost tropoelastin production.

Indeed, it was shown that the efficient translation of mRNAs that promote tolerance to drugs [60] or regulate mitochondrial function [61] depend on the presence of one paralog, but not on that of the other paralogue’s ribosomal protein. Also, in a previous screening study, we found that the ribosomal protein encoded by the RPL35B paralog, but not that encoded by the RPL35A paralog, promotes the increased production level of the Lamb3 protein compromised by a PTC mutation [32]. Moreover, for human ribosomes, for which only a few ribosomal proteins paralogous genes have been reported, preliminary evidence has been obtained that paralogous ribosomal proteins could also serve the specialization of ribosome function [62,63].

This leaves ribosomal protein eL40 as a candidate TRP for boosting tropoelastin 2.7-fold.

This value falls suitably within the range of a two- to four-fold boost of protein production levels, which are reported to result from depletion of ribosomal proteins [32,44], and which represent a change of protein expression level within a moderate and therefore physiological range [45].

### 2.4. Ribosomal Protein eL40 as a Candidate Drug Target for Boosting TE Production

**Figure 4:**
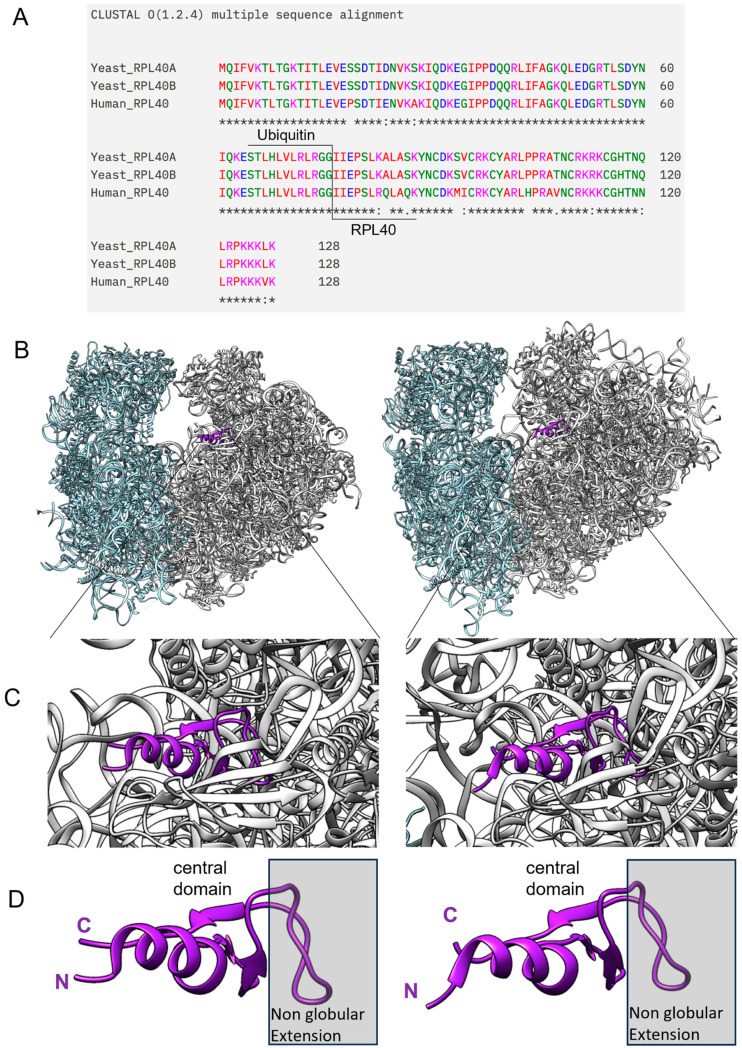
Ribosomal protein eL40 is conserved between yeast and humans in sequence, structure, and topological position on the ribosome. (**A**) Sequence comparison between the two yeast paralogous eL40 proteins, as well as that to their human orthologue, is shown, with the N-terminal ubiquitin tag included. (**B**) eL40 resides in the 60S subunit of eukaryotic ribosomes, yeast shown to the left (PDB code 7B7D) and human shown to the right (PDB code 6QZP), and ribbon models of yeast and human eL40 show their integration into the 60S subunit, with a close-up in (**C**). In (**D**), a visualization of the domain architecture of eL40 depicts the non-globular extension of the protein, which anchors the protein within the rRNA scaffold of the ribosome (grey box). The central domain, as well as the N-terminus and C-terminus, remains accessible for other intermolecular interactions.

Ribosomal protein eL40 is highly conserved in sequence and structure between yeast and humans, as are all 80 of the eukaryotic cytoplasmic ribosomal proteins [37,64,65,66]. Indeed, the comparative sequence alignment of the ribosomal proteins encoded by the yeast paralogous genes RPL40A and RPL40B and by the human single copy gene hRPL40 demonstrates high sequence identity between both the yeast paralogous proteins and the human ortholog (Figure 4A). In addition, both yeast and human orthologs carry an N-terminal ubiquitin tract, which is cleaved off the eL40 core protein before assembly onto the ribosome [67]. On the yeast and human ribosome, rpL40 orthologues occupy identical positions on the 60S subunit (Figure 4B). A closeup of the eL40 protein in yeast and human ribosome (Figure 4C) shows that their respective intrinsically disordered regions are integrated into the rRNA scaffold, as has been described for most other ribosomal proteins [35], while a central region of short beta-sheets and an N-terminal α-helix, as well as a C-terminal tail, remain accessible for other intermolecular interactions (Figure 4D).

**Figure 5:**
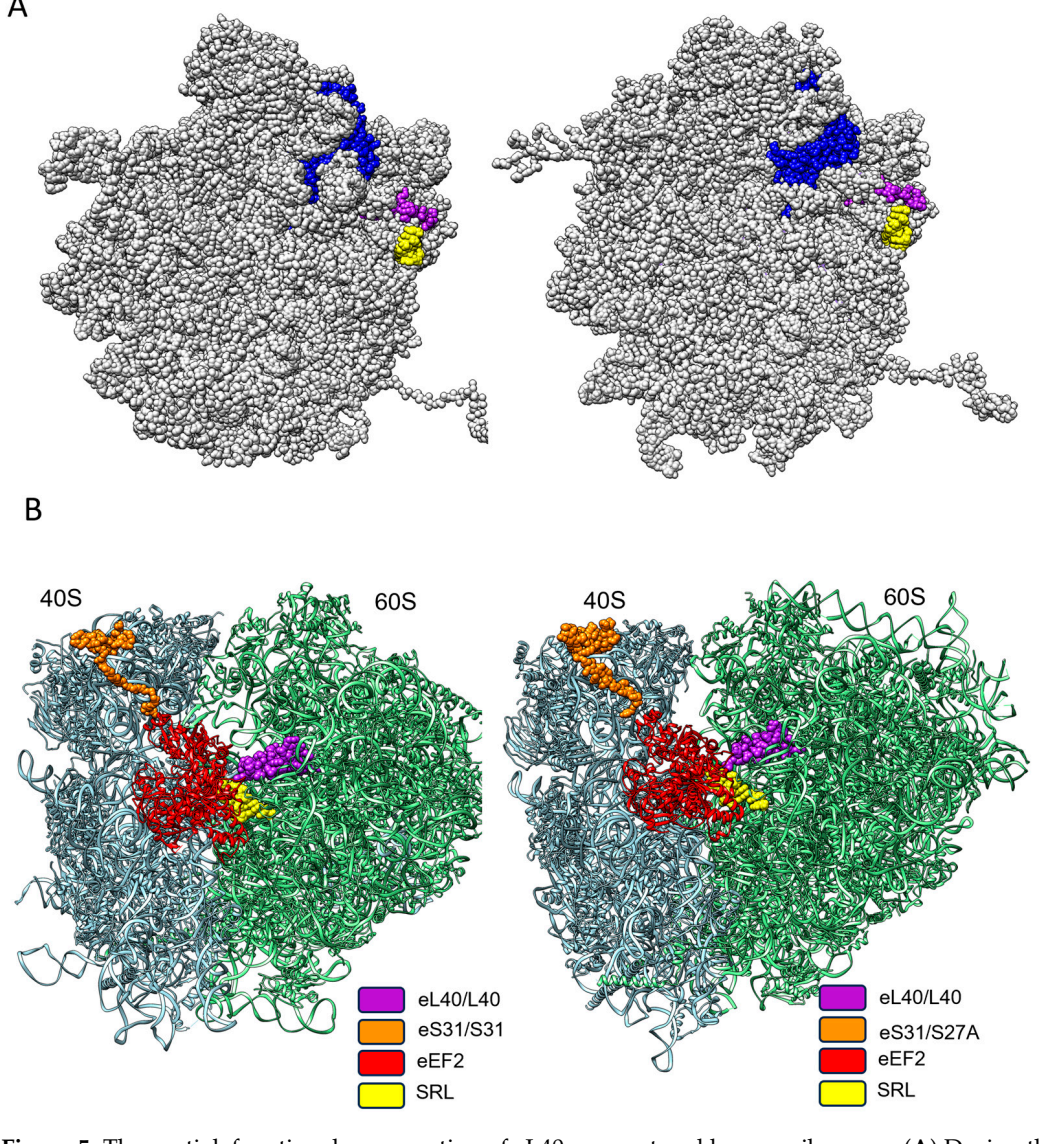
The spatial–functional conservation of eL40 on yeast and human ribosome. (**A**) During the generation of the translation-competent ribosomal subunits, eL40 (blue) is the penultimate ribosomal protein to arrive at the 60S subunit, as shown from the inter-subunit side for yeast on the left (PDB code 7B7D) and for human on the right (PDB code 6QZB). eL40 is positioned atop the Sarcin–Ricin loop (yellow). Upon the arrival of the last ribosomal protein to be incorporated into the 60S subunit, uL16 (magenta), the 60 S subunit, attains its final, translation-competent configuration, which is ready to form the translation-competent ribosome by joining the mRNA-associated 40S subunit. (**B**) On the translation-competent ribosome, yeast to the left (PDB code 5JUU) and human to the right (PDB code 6Z6N) eL40, in close proximity to the small subunit protein eS31 and the Sarcin–Ricin loop of the 60S subunit, respectively, form the landing platform (factor binding site), for elongation factors (EF), which drive the rate of protein synthesis. eEF2, in exemplary form, is shown binding to the factor binding site.

eL40 on the large subunit and ribosomal protein eS31 on the small subunit are the only two eukaryotic ribosomal proteins that carry a single ubiquitin moiety as a ribosomal protein modification [67,68]. Elsewhere, ubiquitination is a common, enzyme-catalyzed, post-translational modification process that adds single-, multi-, or poly-ubiquitin moieties to lysin residues on non-ribosomal substrate molecules. The structurally diverse ubiquitin conjugates direct the substrate proteins to different pathways, for example, proteasomal degradation, endocytosis, histone modification, or the activation of signal transduction pathways [69]. In the case of ribosomal proteins eL40 and eS31, there is evidence that the ubiquitin moiety acts as a chaperone by facilitating their ordered production and folding, as well as timing their coordinated integration into the ribosome, to finally generate the respective translation-competent ribosomal subunits. Indeed, during their final, cytoplasmic maturation, pre-60S ribosomal particles have to integrate deubiquinated eL40 right atop a highly conserved tract of 25S/28S ribosomal RNA (rRNA) of the large ribosomal subunit, termed the Sarcin–Ricin loop (Figure 5A). This process sculpt the 60S inter-subunit site for the acceptance of the ribosomal protein RpL10/uL16, hitherto uL16, the last large subunit RP to arrive at the scene (Figure 5A). Structural studies have shown that eL40 stabilizes rRNA helix 89 to generate the landing platform for uL16. The accommodation of uL16 drives the release of Nmd3, a ribosomal, nuclear export adaptor, which, during 60S subunit maturation, spreads like a protective eagle over the 60S inter-subunit site to prevent untimely ribosomal subunit association [67,68].

Once the multistep translation-initiation process has generated the translation-competent yeast and human ribosome, respectively, eS31 on the small subunit and eL40 on the large subunit, together with the Sarcin–Ricin loop, form the factor binding site, a GTPase activating site. This ensemble forms a ribosomal docking site, which powers GTPase-driven translation factors. Most notably, these are the elongation factor eEF1, which monitors aa-tRNA incorporation as instructed by the mRNA codon sequence, and eEF2, which controls the tRNA translocation during the elongation of the protein synthesis (Figure 5B) [70].

Given the amazing consensus on the structural and functional parameters of yeast and human eL40, we conclude that eL40, the yeast ortholog identified by RiboScreen^TM^ technology as a ribosomal switch to boost tropoelastin expression, might also be explored as a human target ribosomal protein (TRP) for the discovery of small-molecule ligands.

### 2.5. In Silico Screen Identifies eL40 Hit Molecules

The next step of RiboScreen^TM^ technology platform requires the identification of candidate ligands to the target ribosomal protein (TRP) eL40 for boosting the production levels of tropoelastin. Previously, yeast rpL35/uL29 was identified based on the screening of an RVS library, hitherto L35, as a TRP for boosting the production levels of full-length skin protein Lamb3. By serendipity, a biotinylation assay had delivered Artesunate as a putative ligand of human L35 [71]. We then were able to show that treatment with Artesunate in naïve yeast vehicles [34] and in human HaCat cells [46] triggered a dose-dependent increase in production levels of full-length Lamb3. However, to date, there are no reports in the literature on possible yeast or human eL40 ligands. Therefore, as RiboScreen^TM^ delivered eL40 as a functionally defined target for drug discovery, we opted for a computational, virtual screening approach.

**Figure 6:**
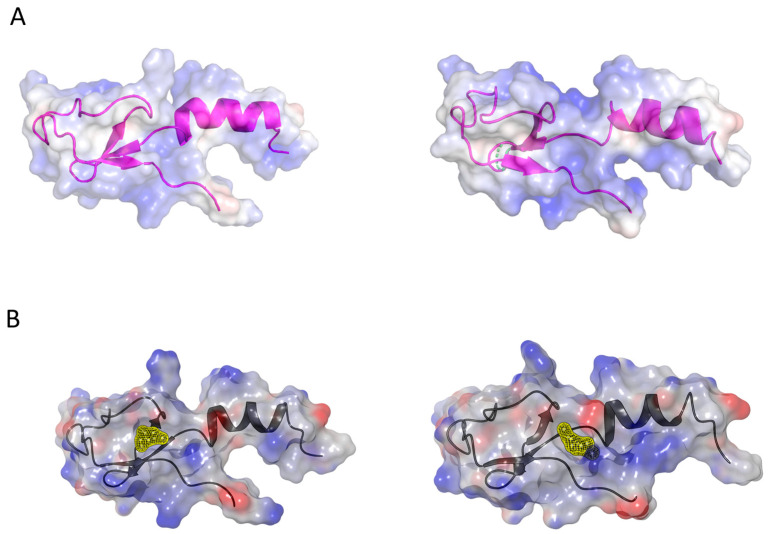
Ligand binding sites of eL40 and the visualization of binding pockets. (**A**) Coulombic surface representation of yeast (left, PDB code 3J77) and human (right, PDB code 6XA1) eL40 proteins, overlaid on their secondary structures. (**B**) Potential small-molecule binding pockets on yeast eL40, to the left, (yellow, near Ser94, PDB code 3J77) and human eL40, to the right (yellow, near Asp92, PDB code 6EK0), were identified by druggability analysis (Sitemap [72]) using the amino acid sequence of the eL40 proteins from the ribosome X-ray structures (minus rRNA). These pockets are adjacent (yellow patches) and, depending on protein dynamics, could potentially form a larger, common binding cavity.

First, employing the Chimera program suite, we calculated the Coulomb surface potential of yeast and human eL40. Such a surface potential analysis allows for the visualization of areas on the protein surface where there are potential favorable electrostatic interactions with charged or polar ligands. The inspection of Coulombic surface of yeast and human eL40 proteins, superimposed on their secondary protein structure, suggests a possible ligand binding site in the center of the rpL40/eL40 protein, a domain that, in the assembled ribosome, is not covered by an rRNA scaffold and that forms the base for the other accessible extensions of eL40, the N-terminal helix, and the C-terminal tail (Figure 6A). This central domain harbors a cavity lined by basic amino acids, which would provide a docking site for acidic or polar small molecules. 

In a reductionist approach, the amino acid coordinates of the eL40 protein are extracted from the ribosome X-ray structure in the absence of rRNA. This simplified representation of the eL40 protein is used as model structure for a druggability and docking analysis. The eL40 protein is composed of 52 amino acids. A druggability analysis using SiteMap [72] revealed a small pocket in humans near amino acid Asp92 (PDB code 6ek0) and in the yeast structure near amino acid Ser94 (PDB code 3J77). Both pockets have low druggability scores of 0.4 and are adjacent to each other. The comparison of binding sites of yeast and human structures, identified by Sitemap, shows that the binding sites with the highest druggability score are adjacent to each other (yellow patches in Figure 6B), and we speculate that a common larger pocket could be formed depending on protein dynamics.

Next, a bioinformatic approach was taken to identify possible eL40 ligands. Therefore, a subset of Merck’s in-house library was virtually screened using Glide docking to identify small molecules that could potentially bind into the pocket. A visual inspection and analysis of docking scores revealed 33 putative eL40 binders. These 33 eL40 ligands can be grouped into four major clusters: carboxylic acids, peptides, natural compounds and novel chemical entities (NCE). In exemplary form, representatives of the hit molecules that are filed for patent protection (WO24099952 A1) will be represented in more detail below.

### 2.6. In Cell Assays Identify RpL40 Ligands That Boost Production Level of Tropoelastin

The next step in RiboScreen^TM^ technology is to test putative eL40 ligands in cellular assays for their activity in editing TRP eL40 for a boost in tropoelastin production. Given the evolutionarily highly conserved structural and functional homology of yeast and human ribosomal proteins eL40, as well as the presence of identical predicted ligand binding sites (Figure 4 and Figure 6), a cost-saving and effective strategy to test the functional activity of human eL40 ligands is provided by testing the candidate small molecules in naïve yeast vehicles. In this scenario, the wild-type ribosome complement is present, and a potential manipulation of TRP eL40 function by small-molecule ligand is monitored by recording the changes in the TE-FF reporter protein production levels. 

Accordingly, 29 putative candidate eL40 small-molecule binders, which, out of the set of 33 hits, were available for screening, were assayed in naïve vehicles harboring the TE-FF reporter and the REN companion reporter. This is the same dual luciferase reporter pair that was employed in the first step of the RiboScreen^TM^ technology, where the screening of the RVS collection delivered eL40 as a ribosomal switch to increase the production levels of tropoelastin. Here, for the experiments testing the activity of the eL40 small-molecule ligands, we employed the Promega Dual Glow Assay. In comparison to the Promega dual luciferase assay used for screening the RVS library, this allows for faster, parallel processing of luciferase signal readouts when monitoring in-yeast vehicles over a range of concentrations, the effect of small-molecule treatment on the change of production level of a reporter protein.

**Figure 7:**
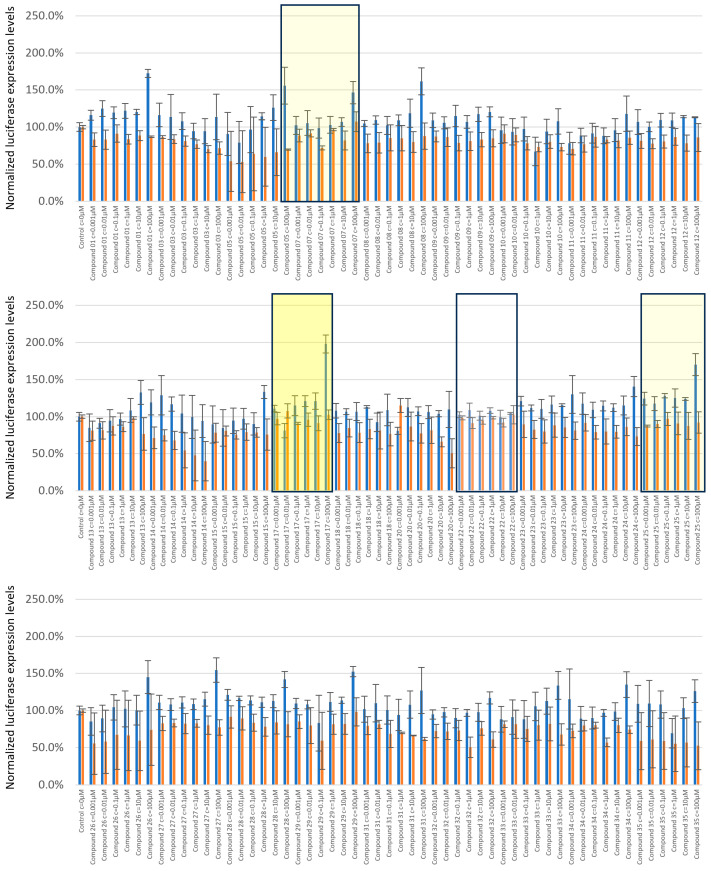
Validation of small-molecule eL40 ligands for customized boost of tropoelastin production levels. Candidate small-molecule ligands were tested in a range of concentrations from 1 nM to 100 µM. Firefly tagged tropoelastin luciferase signals (blue) and Renilla signals (orange) obtained upon treatments are shown normalized to the untreated control. The compounds are listed per increasing number, and C17 showed a dose-dependent and significant response at 100 μM, with a 1.7-fold boost in tropoelastin reporter expression, and Renilla expression was unaltered (middle panel, yellow rectangle). Examples of minor candidate eL40 activators are C7 and C25 (mute yellow). A representative of non-active eL40 ligands is C22 (lower panel, white).

Small-molecule ligands were tested in a range of concentrations from 1nM to 100µM. Luciferase signals were recorded, filed, and subjected to statistical analysis (Supplementary Table S2). Of the 29 candidate molecules tested, compound 17 (C17) showed a dose-dependent and significant response at 100 µM with an increase in TE-FF reporter expression levels at 1.7-fold, with REN control expression levels unaltered (Figure 7, first panel). Interestingly, this 1.7-fold increase in TE reporter protein production level mirrors the similar 2.7-fold increase delivered by that RVS screening vehicle, which is demarcated by a depletion of eL40 and therefore defined rpL40 as the candidate TRP for a boost of tropoelastin.

C17 is the most promising compound, as increased expression levels of tropoelastin are accompanied by unaltered protein production levels of the companion reporter protein REN (Figure 7, Supplementary Table S2). We also found a few hit molecules with a more minor potential to boost tropoelastin production, and where the expression levels of companion reporter REN were also altered (Supplementary Table S2). These are exemplified by compound 7 (C7) and compound 25 (C25) (Figure 7). The majority of the tested compounds showed no or little response to altering either TEFF or REN production levels, with compound 22 (C22) yielding protein production levels identical to untreated vehicles at all concentrations used (Figure 7). 

**Figure 8:**
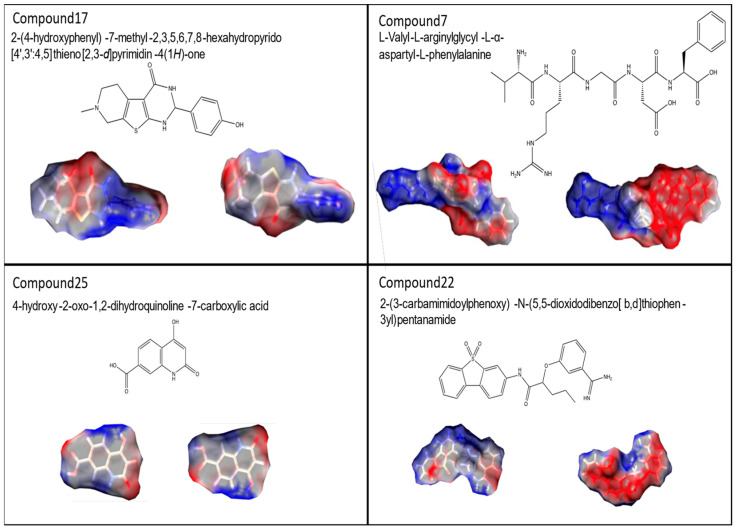
Molecular structures of rpL40 ligands. UPAC nomenclature, molecular structure and space filling models of compounds C17, C7, C25, and C22 are shown.

Here, we present in more detail the molecular parameters of hit compound C17, minor species C7 and C25 and non-active compound C22, all of which have been filed for patent protection (WO24099952 A1) (Figure 8).

**Figure 9:**
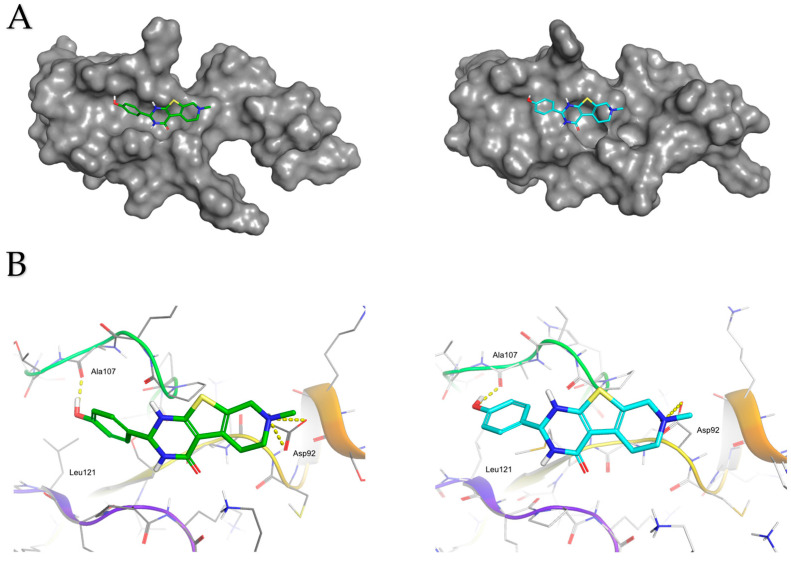
Molecular interactions of C17 with yeast and human rpL40. (**A**) Predicted docking poses for C17 with yeast (PDB code 3J77) and human (PDB code 6EK0) eL40 structures. This figure illustrates the predicted binding modes of compound C17 to both yeast and human eL40 orthologues. On the left, the complex structure with the yeast eL40 is shown, where the carbon atoms of C17 are highlighted in green. On the right, the complex structure with the human eL40 is displayed, with C17’s carbon atoms in cyan. The molecular surfaces of both structures are depicted in panel (**A**). Panel (**B**) highlights the key interactions: the charged interactions between the piperidine group of C17 and the aspartate 92 residue, and the hydrogen bond between the phenolic group of C17 and the backbone of alanine 107.

We wished to characterize the binding mode of C17 to eL40 in more detail. This showed that C17 adapts a flat shape and, in the docking pose the phenolic group, makes a hydrogen bond interaction with the backbone oxygen of alanine 107 in human and yeast (Figure 9A,B). The piperidine moiety of the tricyclic core makes a charged interaction with aspartate 92 in humans and yeast. The binding mode is predicted to be similar in yeast and human structure. For the inactive C22, we do not observe a consistent binding mode to eL40 protein.

The use of in silico docking virtual screening to identify potential eL40 ligands is a notable achievement in this study. Virtual screening is a powerful computational technique that enables the rapid evaluation of large chemical libraries against a target protein structure. It prioritizes the compounds that are most likely to interact with the target based on their predicted binding modes and scores.

Using the three-dimensional eL40 structure and employing docking simulations with Merck’s in-house compound library, we were able to efficiently identify 33 putative binders to the eL40 pocket. The Merck library has been docked using Glide SP (version 8.3) mode with standard settings and the top 200 scoring molecules have been prioritized using visual inspection. Besides the Glide docking score, emphasis was put on predicted docking poses that are in the predicted pocket, and docked poses interact with amino acids alanine 92 and aspartate 107. An important aspect of this approach is the use of a reductionist representation of eL40. Despite working with an isolated protein subunit structure, which contained only a subset of information compared to the full ribosomal context, the docking procedure was able to identify potential binders. 

After a functional assay to test the activity of the 29 eL40 binders, which were physically available for treatment studies, and in combination with Glide docking followed by visual inspection, we were able to identify and characterize 3 binders out of the 29 tested molecules, resulting in a high hit rate. This underscores the effectiveness of the computational strategy employed, even for a challenging target like eL40 with its relatively small and shallow binding pockets.

In sum, this highlights the robustness of the virtual screening approach and its ability to capture essential features of the binding pocket. The successful identification of hit compounds, such as C17, which exhibited dose-dependent activity in boosting tropoelastin production levels, validates the utility of this computational strategy. This approach not only accelerates the discovery process but also reduces the associated costs and resources required for traditional screening methods.

We wanted to better understand the activity of L40 binders and their accessing of the yeast and human eL40 proteins, which led us to revisit eL40 in the assembled ribosome. First, we observed that, with respect to the entry into the assembled ribosome, the active small molecules reported here are all within a molecular weight (MW) of 300 to 500 Daltons. C17 has an MW of 315, C7 has an MW of 592, and C22 has an MW of 449. Therefore, they could in principle access and bind to eL40 ribosomal protein domains not immersed in the rRNA scaffold. Comparative inspection of Figure 4 and Figure 6 suggests that the delivery of small-molecule ligands is possible for a central domain that extends out from the rRNA scaffold into the ribosomal inter-subunit space. Such modes of entry into the assembled ribosome have been studied for rRNA binding small molecules, most notably aminoglycoside antibiotics [73], which are of similar molecular size compared to the active eL40 ligands described in this study.

Second, our analysis revealed a recurring feature in the active RpL40 small ligands: opposing patches of high and low electron density on these relatively flat molecules. This characteristic could be responsible for two synergistic functionalities. On the one hand, the regions with a lower electron density (a more positive character), in concert with other structural features, might aid in the molecular recognition and facilitate an approach by being repelled from areas of positive (partial) charge on other ribosomal proteins, thereby preventing non-specific binding. On the other hand, the patches with a higher electron density (a more negative character), along with meeting the size and shape requirements for the central eL40 binding pocket, could determine the specific interaction between the small molecule and the ribosome. 

Third, we asked in which way the binding of small molecules to a ribosomal protein could trigger a customized increase of the tropoelastin reporter protein. For rRNA-binding small molecules, as exemplified by Aminoglycoside antibiotics that bind to the mRNA decoding site (A-site) of the assembled ribosome, it is known that their binding reconfigures the A-site with the functional consequence of changing the fidelity and elongation rates of the translating ribosome [74,75].

Although it is well established that the binding of a small molecule to a protein changes the function of a target [76], where small changes in conformation may result in significant alterations in binding affinities of the target protein to partner molecules, at present, it is not known how ligand binding could alter the ribosomal protein function in the translation of distinct mRNAs. It has been speculated that each of the 80 ribosomal proteins in a cell type or metabolic-state-dependent fashion, fine-tunes the protein production level of only a selected set of proteins [44,45,77]. In the case of eL40, the binding of candidate C17, as well as the binding of the minor candidate small molecules, could either alter eL40 anchoring to the ribosome or alter the affinity of such ribosome binding proteins that dock onto the ribosome close to eL40, for example, translation elongation factors [70]. In the latter case, a possible role of eL40 as a ribosomal regulator of elongation rates of translation might be targeted. As noted above, eL40 contacts the Sarcin–Ricin loop, and together with S31, this molecular triad forms the docking site for the elongation factors that drive protein elongation (Figure 5), [67,68]. We hypothesize that the binding of hit small molecules to eL40 modulates the interaction of this ribosomal protein with elongation factors so that elongation rates are favorably altered for the boost of TE production levels.

## 3. Materials and Methods

### 3.1. Yeast Strains

In the yeast S. cerevisiae, the 78 RPs are encoded by 137 genes, which include 19 single copy RP genes and 59 duplicated RP genes. A total of 128 different diploid strains, heterozygous for deletions of one or the other of the RP genes (variant ribosomal protein strains) and viable under the culture conditions used in this study, were obtained from the EUROSCARF gene deletion collection [78,79]. The remaining 9 strains could not be revived from long-term storage. Yeast and E. coli media, culture conditions, and the manipulation of yeast and E. coli strains were as described previously [80]. The employed RP deletion strains were grown on yeast-extract–peptone–dextrose (YPD) medium or defined yeast nitrogen base (YNB)-based medium/synthetic complete (SC) medium to provide the desired selective conditions.

### 3.2. Cloning of the Tropoelastin–Firefly Protein Expression Reporter

The reporter parent vectors used were the yeast centromeric plasmids YCplac33 (URA3) and YCplac111 (LEU2) [81]. To generate the tropoelastin-FF Luciferase reporter, first, the tropoelastin sequence (tropoelastin isoform VI) was PCR-amplified from the parent vector pCMV6-XL6_ELN-Iso-VI [52] using the forward primer F 5’-GGACTCTAGAGCGGGTCTGACGGCGGC-3’ and the reverse primer R 5’-CCTGGGTACCTTTTCTCTTCCGGCCACAAG-3’, containing restriction sites for XbaI at the 5’ and KpnI at the 3’ end. A previously generated YCpLac33_LAMB3-PTC_FF plasmid [32], which was generated to express the reporter sequence under the control of ADH1 Promotor and ADH1 terminator sequences, respectively, was digested with FastDigest XbaI [82] and FastDigest KpnI [82], Thermo Scientific, Linz, Austria, to replace the LAMB3-PTC sequence with tropoelastin sequence. This delivered the TE_FF reporter. The correct integration of the tropoelastin sequence was verified by sequencing and a control digest. The Renilla (REN) protein expression reporter, also under the control of and ADH1 Promotor and ADH1 Terminator, respectively, was available from previous studies [32] and harbors the Renilla sequence in a YCpLac111 parent vector [81].

### 3.3. Dual Luciferase^®^ Reporter Assay in Ribosomal Protein Variant Library Screening

Diploid wild-type (BY2n) and diploid variant ribosomal protein yeast strains, the ribosomal protein variant (RVS) screening library [32,53,78], were co-transformed with the tropoelastin–Firefly and the Renilla protein expression reporter plasmids at 1μg/μL each, following our established protocol [32]. Single colonies of transformed vehicles were used to generate overnight cultures of wild-type and RVS vehicles. Cell density (OD600) was determined using a Hitachi U_ 3000 spectrophotometer. Erlenmeyer flasks containing 13 mL SC-URA-LEU medium were inoculated at OD600 = 0.065. Cultures were grown overnight at 28 °C with constant shaking (250 rpm) and then harvested near the exponential phase, at OD600 = 1.8 to 2.0 and centrifuged, and the pellet was brought to 107 cells mL1 in autoclaved water. Dual luciferase readouts were recorded on the GloMax Multi Detection device. For each dual luciferase luminescence, a 50 µL measurement of a cell culture (5 × 105 cells) was transferred into a 96-well plate (Perkin Elmer, Black and White Isoplate, 1450-582) and lysed by the addition of 20 µL passive lysis buffer (Promega) for one minute, following the instructions of the supplier. The FF luminescence readout was detected by the addition of 50 µL of LAR II substrate, and then the REN luminescence signal was recorded by subsequent addition of 50 µL Stop & Glo^®^ substrate.

To monitor any possible changes in protein production levels of the co-expressed Luciferase reporter pair in the RVS screening vehicles, the luminescence readouts of the companion TE-FF and REN reporters in wild-type cells were optimized and validated to show the functionality of the protein reporter pair. Using the Promega Dual luciferase system, in wild-type vehicles, the mean luminescence readout of TE-FF reporter protein expression when co-expressed in the presence of the companion REN reporter was 1.0 × 10^4^ luciferase light unit counts, and that of the REN reporter was 8.0 × 10^7^ luciferase light unit counts. Such a difference in luciferase signal strength between REN and FF reporters, where the FF reporter is coupled to a large, heterologous human protein, has been reported previously [32,83]. Also, luciferase signals in wild-type cells, for both the TE-FF and the REN reporter, are significantly higher than the limit of quantification, by one order of magnitude for the Firefly recordings and three orders of magnitude for the Renilla recordings. This ensures that the screening tool is able to cover the effects of a given ribosomal protein modifications on change in production levels of a selected reporter protein, which is reported to be in the range of 2- to 4 -fold up and to 2- to 4-fold down [32,44].

For this screen, two biological replicates per variant ribosomal protein yeast strain, each resulting from independent yeast transformations, were measured in 3 technical replicates each to deliver at least 6 luciferase signal readouts for the assessment of the production levels of TE-FF and REN, respectively. The expression levels of TE-FF and REN reporters in wild type cells were recorded in parallel. The raw data of luciferase readouts of RVS vehicles were recorded as CSV files, and data were further processed as described in the statistics section.

### 3.4. Dual Glow^®^ Luciferase Assay in Semiautomated 96-Well Assay for Small-Molecule Screening

Wild-type yeast strains were co-transformed with the TE-FF reporter plasmid and REN reporter plasmid at 1 μg/μL each. Seventy-two hours after transformation, a single colony is inoculated overnight in 3 mL of selective media (SC-U-L). Then, cells from the overnight culture are inoculated in 3 mL SC-U-L (OD600 0.2) and grown to an exponential phase at 28 °C, 250 rpm for 7 h. After this, they are diluted to an OD600 of 0.004 in 25mL and dispersed in equal amounts (198 μL) in a 96-well cell culture plate. For treatment, for each well, 22 μL of the small-molecule solution of appropriate concentration was added to cover a range of 1 nM to 1μM treatment, and in the case of control condition, 22 μL of medium was added. The 96-well plate was then covered with a lid, placed in a Styrofoam box equipped with a water reservoir to avoid evaporation, and incubated for 18 h at 28 °C at 250 rpm. 

Afterwards, cells were harvested at an optical density OD600 = 1.5, and measurements were performed in triplicate, using the Dual-Glo^®^ Luciferase Assay System to monitor Firefly and Renilla luminescent signals. A total of 50 μL of cells from each culture condition were transferred into a 96 black-and-white well plate (PerkinElmer), and to each well, 50 μL of Dual-Glo substrate was added and mixed properly, and the plate was incubated for 20 min (to allow lysis to occur as Dual-Glo substrate also contains lysis buffer). After incubation, Firefly signals were recorded and 50 μL of Dual-Glo^®^ Stop & Glo^®^ substrate was added, and after 20 min incubation, Renilla signals were recorded. 

To establish the functionality in the Dual Glo luciferase assay of the luciferase reporter, proteins in untreated wild type vehicles, luminescence readouts of the companion TE-FF, and REN reporters were recorded. The mean luminescence readout of the TE-FF reporter protein expression when co-expressed in the presence of the companion REN reporter was 2.5 × 10^3^ luciferase light unit counts, and that of the REN reporter was 1.7 × 10^5^ luciferase light unit counts. The signal strength of both the heterologous Firefly reporter protein and the Renilla reporter agree with what has been reported by the supplier (https://at.promega.com/resources/guides/cell-biology/bioluminescent-reporters, accessed on 13 June 2022). Th recodings of the Firefly and Renilla luciferase signals upon small-molecule treatment were stored as CSV files and transferred into Excel 2016 files for statistical processing.

### 3.5. Statistical Analysis

For the analysis of RiboScreen^TM^ data, the reporter protein luciferase readout data obtained for the TE-FF reporter and the REN reporter in each ribosomal variant strain and wild type vehicle, respectively, were recorded utilizing the GloMax Multi Detection device. Initially, the reporter luciferase readouts from the various ribosomal protein deletion strains were subjected to outlier detection by employing the Grubbs test. This statistical method operates under the assumption that the collected data are drawn from a larger normal distribution. Any identified outliers were duly marked and subsequently excluded from further analysis to ensure data integrity. Subsequently, the data sets underwent a comprehensive descriptive statistical analysis aimed at determining the mean and standard deviation for each individual data set. The statistical evaluations were conducted using MiniTab 21 and Microsoft Excel, both of which are robust tools for data analysis. The differential expression levels of the reporter proteins across the variant ribosomal protein deletion strains were visualized through the application of the statistically analyzed data sets, thereby enhancing the interpretability of the results. In a parallel approach, the luciferase readout data obtained from small-molecule treatments of wild-type yeast vehicles were processed using statistical tools as described above. This ensured consistency in the analytical framework applied to both analysis of reporter protein expression levels in the variant ribosomal protein deletion strains, as well as in the wild-type vehicle treatment groups, thereby facilitating a comprehensive understanding of the underlying biological phenomena.

### 3.6. In Silico Studies

The programs Chimera [84] and Pymol [85] were employed for the visualization of yeast and human ribosome and ribosomal proteins. The eL40 protein’s 3D structures from the Protein Data Bank (PDB codes 3J77 and 6EK0) were optimized using Maestro’s Protein Preparation Wizard (Schrödinger, LLC) to enhance hydrogen bonding networks and assign protonation states at a physiological pH. SiteMap [86] was used to assess potential binding sites. The Inhouse Merck compound library has been prepared using LigPrep with standard settings. Glide (Schrödinger, LLC) [87] facilitated docking simulations, and pose selection criteria involved key protein–ligand interactions, binding energy estimations, and visual inspection. Cluster analysis identified four major clusters: carboxylic acids, peptides, natural compounds, and novel chemical entities (NCE). Caco-2 permeability and cytotoxicity in a HepG2 cell line were evaluated via predictions of the respective machine learning models as integrated into the software platform AIDDISON [88].

## 4. Conclusions

RiboScreen^TM^ technology is a platform technology that initially employs yeast vehicles as a genetic and then a chemical screening platform to identify hit small-molecule ligands of yeast and human target ribosomal proteins to drive changes in protein production levels of selected proteins of interest. In this study, we deliver small molecules, which, in virtual docking studies, were shown to bind to both yeast and human L40e and demonstrate that the treatment of naïve yeast vehicles with these hit molecules boosts tropoelastin production levels up to two-fold. This sets the stage for studying the small-molecule hits for their activity in human cell models. This will first warrant the testing of the combinatorial use of these hit molecules first in yeast and then in human cells and, given a synergistic effect, might deliver a reduction in the concentration of either small molecule used for treatment, as was observed to be the case in our previous study [34]. Second, lead optimization might deliver hit molecule derivatives with better efficacy. Third, proteomic and immunofluorescence techniques will have to monitor drug-induced increases in tropoelastin production in human fibroblasts to assess the functionality of this intervention. 

## 5. Patents

The patent family represented by WO24099952 A1 based on EP4368729 is filed in the name of Merck Patent GmbH.

## Data Availability

Data are provided in the Supplementary Materials of this report.

## Supplementary Materials

The following supporting information can be downloaded at https://www.mdpi.com/article/10.3390/ijms25158430/s1.

## Abbreviations

ECM	Extracellular matrix
UV light	Ultraviolet light
TRP	Target ribosomal protein
RVS	Ribosomal protein Variant Strain
eL40	Ribosomal protein L40
RP gene	Ribosomal protein gene
YPD	Yeast peptone dextrose
YNB	Yeast nitrogen base
ADH1	Alcohol dehydrogenase 1
LAMB3-PTC	Gene encoding laminin beta 3 subunit harboring a premature termination codon (PTC) mutation
TE-FF	Tropoelastin C-terminally tagged with fire- fly luciferase reporter
REN	Renilla luciferase reporter
POI	Protein of interest
rRNA	Ribosomal ribonucleic acid
ELN	Human gene encoding tropoelastin
3′UTR	3′untranslated region
AP1	Activator Protein 1
GR	Glucocorticoid Receptor
GRE	Glucocorticoid Response Elements
Fox01	FoX01 gene
FRE	Focused Regulatory Elements
EGF	epidermal growth factor
HB-EGF	Heparin-Binding EGF-Like Growth Factor
TNF	Tumor necrosis factor α
CAAT box	Regulatory eucaryotic promotor element
TGF-α	Transforming Growth Factor Alpha
bFGF	basic Fibroblast Growth Factor
JUN	gene encoding c-Jun
IL1β	interleukin 1 β
PDGF	Platelet-Derived Growth Factor
IGF1	Insulin growth factor 1
Sp1	Specificity Protein 1,
Rb	Retinoblastoma protein
GC box	Regulatory eucaryotic promotor element
C/EBPβ	CCAAT/enhancer-binding protein beta
NF-κB	Nuclear factor kappa-light-chain-enhancer of activated B cells
CTCF	CCCTC-binding factor
