# Supplementary figures and images for: RiboScreenTM Technology Delivers a Ribosomal Target and a Small-Molecule Ligand for Ribosome Editing to Boost the Production Levels of Tropoelastin, the Monomeric Unit of Elastin
Source: Int J Mol Sci. 2024 Aug 1;25(15):8430. doi: 10.3390/ijms25158430 (PMC11312584; doi:10.3390/ijms25158430)

## Slide 1
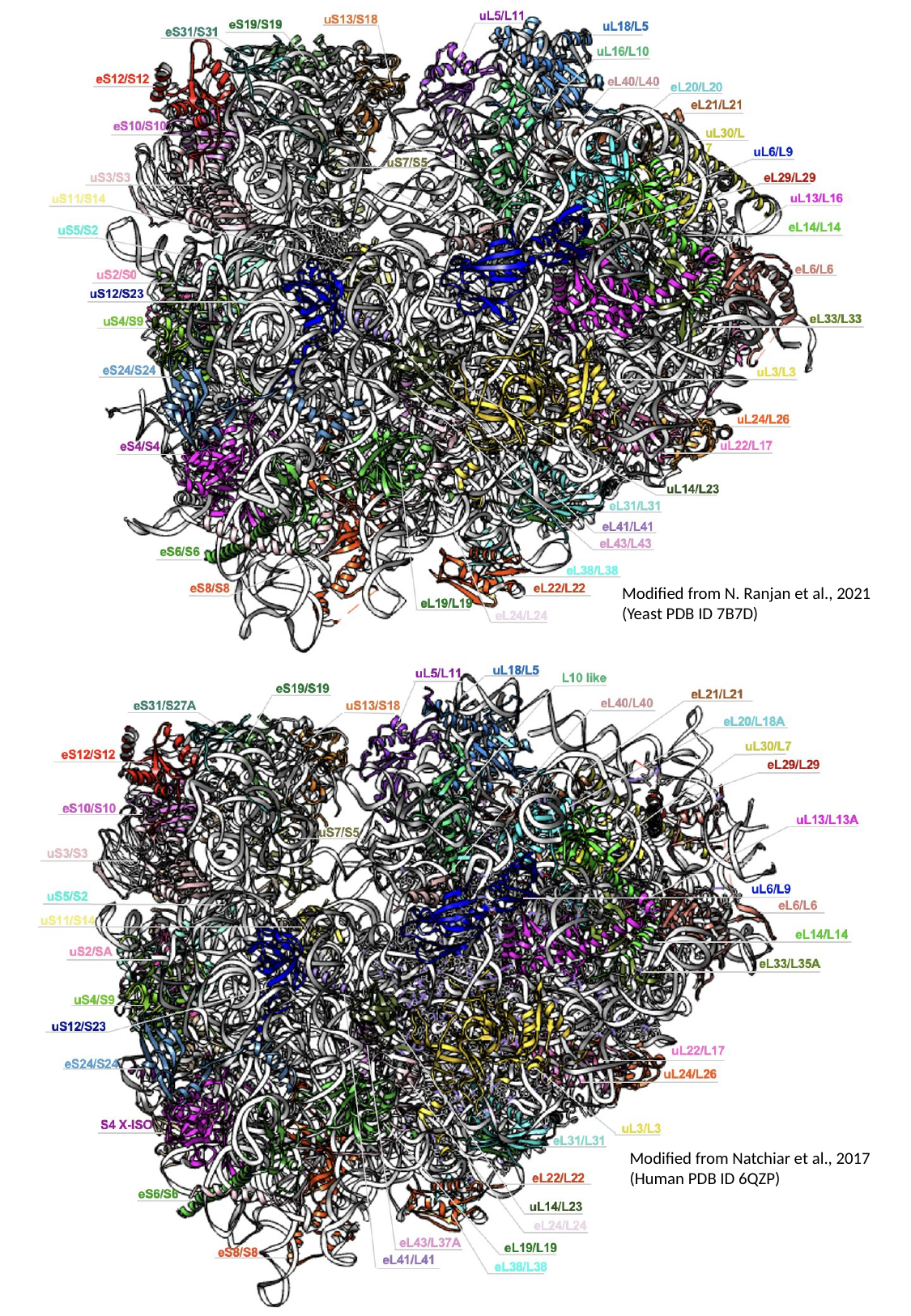

Modified from N. Ranjan et al., 2021
(Yeast PDB ID 7B7D)
Modified from Natchiar et al., 2017 (Human PDB ID 6QZP)

Supplement: Supplementary file 1 [file ijms-25-08430-s001.zip › Supplementary_Figure_01.pptx]
